# Oligomeric self-association contributes to E2A-PBX1-mediated oncogenesis

**DOI:** 10.1038/s41598-019-41393-w

**Published:** 2019-03-20

**Authors:** Chiou-Hong Lin, Zhong Wang, Jesús Duque-Afonso, Stephen Hon-Kit Wong, Janos Demeter, Alexander V. Loktev, Tim C. P. Somervaille, Peter K. Jackson, Michael L. Cleary

**Affiliations:** 1 Department of Pathology, Stanford University School of Medicine, Stanford, CA 94305 USA; 2 Department of Hematology and Oncology, University Medical Center Freiburg, Freiburg, Germany; 3 Baxter Laboratory for Stem Cell Biology, Department of Microbiology and Immunology, Stanford University School of Medicine, Stanford, CA 94305 USA; 4 Present Address: Sun Yat-Sen University, School of Pharmaceutical Sciences, Guangzhou, 510006 China; 5 Present Address: Leukaemia Biology Laboratory, Cancer Research UK Manchester Institute, The University of Manchester, Manchester, M20 4GJ UK

## Abstract

The PBX1 homeodomain transcription factor is converted by t(1;19) chromosomal translocations in acute leukemia into the chimeric E2A-PBX1 oncoprotein. Fusion with E2A confers potent transcriptional activation and constitutive nuclear localization, bypassing the need for dimerization with protein partners that normally stabilize and regulate import of PBX1 into the nucleus, but the mechanisms underlying its oncogenic activation are incompletely defined. We demonstrate here that E2A-PBX1 self-associates through the PBX1 PBC-B domain of the chimeric protein to form higher-order oligomers in t(1;19) human leukemia cells, and that this property is required for oncogenic activity. Structural and functional studies indicate that self-association facilitates the binding of E2A-PBX1 to DNA. Mutants unable to self-associate are transformation defective, however their oncogenic activity is rescued by the synthetic oligomerization domain of FKBP, which confers conditional transformation properties on E2A-PBX1. In contrast to self-association, PBX1 protein domains that mediate interactions with HOX DNA-binding partners are dispensable. These studies suggest that oligomeric self-association may compensate for the inability of monomeric E2A-PBX1 to stably bind DNA and circumvents protein interactions that otherwise modulate PBX1 stability, nuclear localization, DNA binding, and transcriptional activity. The unique dependence on self-association for E2A-PBX1 oncogenic activity suggests potential approaches for mechanism-based targeted therapies.

## Introduction

Fusion proteins with the features of chimeric transcription factors are frequently created by chromosomal translocations in acute leukemia^1^. Generation of these diverse factors is typically an early, initiating event in acute leukemogenesis with important patho-biological and clinical implications. Although the complete mechanisms underlying their oncogenic effects are not fully understood, one emerging theme is their ability to self-associate into higher-order molecular complexes^2–4^, reminiscent of the role of constitutive self-association in the oncogenic activation of chimeric kinases^5^. However, the specific roles, if any, for oligomerization in activating the transcriptional and leukemogenic properties of most chimeric transcription factors remain poorly defined.

The PBX1 proto-oncoprotein is a TALE (three amino acid loop extension) class homeodomain protein, which is a component of hetero-oligomeric transcriptional complexes that regulate developmental gene expression^6–8^. Lack of PBX1 results in embryonic lethality and several embryonic defects partially phenocopy loss of various HOX, MEINOX, or orphan homeodomain proteins^9,10^, consistent with the *in vitro* properties of PBX1 as a DNA binding cofactor for a large subset of homeodomain transcription factors with roles in multiple developmental programs^11–13^.

PBX1 is converted into a chimeric transcription factor by t(1;19) chromosomal translocations in about 5% of pediatric and adult acute lymphoblastic leukemia and (rarely) myeloid leukemia^14,15^. It is oncogenically activated by in-frame fusions with E2A (also known as TCF3) proteins, which are transcriptional regulators of the bHLH family with critical roles in the development and differentiation of several cellular lineages^16,17^. Fusion with E2A dramatically alters the biochemical and transcriptional properties of PBX1, with likely impacts on both the E2A and PBX1 subordinate pathways. The E2A moiety confers strong transcriptional activator and constitutive nuclear localization properties on E2A-PBX1^18^, which retains the PBX1 homeodomain DNA binding motif. This gain of function, which is critical for oncogenic activity, abrogates interactions with and dependence on MEINOX homeodomain proteins, which conditionally regulate the stability, nuclear import and cooperative DNA binding of wild type PBX1^19–21^. Chimeric E2A-PBX1 oncoproteins retain an ability to bind DNA in association with HOX transcription factors^22,23^, and co-expression with HOXA9 accelerates E2A-PBX1 mediated leukemogenesis^24^, suggesting that E2A-PBX1 directly impacts the HOX transcriptional regulatory pathway in acute leukemia pathogenesis. A provisional molecular model proposes that E2A-PBX1 perturbs the expression of critical subordinate genes as a simple heterodimeric complex with HOX DNA binding partners^22,24^. However, HOX gene expression profiles in E2A-PBX1^+^ ALL cells are highly variable^25,26^ and most of the supporting experiments employed forced expression of HOX genes^24,27^, raising the possibility that undefined pathways may contribute to E2A-PBX1 leukemogenesis.

We report here that E2A-PBX1 self-associates through the PBC-B domain of the chimeric protein to form higher-order oligomers in t(1;19) human leukemia cells. Self- association is required for oncogenic activity and facilitates the binding of E2A-PBX1 to DNA. These studies suggest a revised model for E2A-PBX1 leukemogenesis in which self-association compensates for its inability to stably bind DNA or dimerize with heterologous MEINOX protein partners that otherwise regulate PBX1 DNA binding and transcriptional activity.

## Results

### Identification of E2A-PBX1 interacting proteins using LAP-tag purification

**Figure 1:**
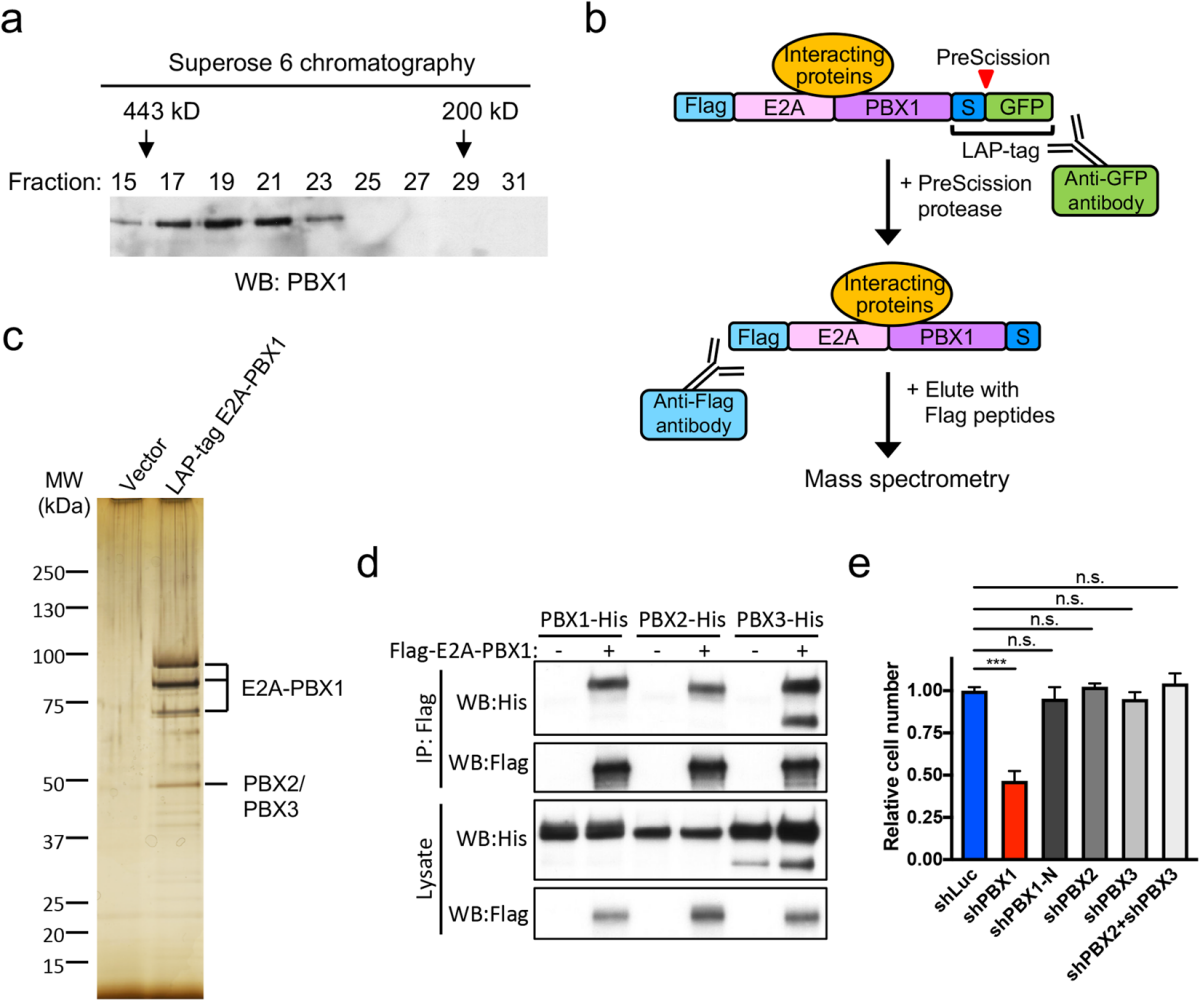
Identification of E2A-PBX1 interacting proteins using LAP purification. (**a**) Superose-6 gel filtration analysis was performed on a protein extract of the RCH-ACV E2A-PBX1^+^ leukemia cell line. Each column elution fraction was fractionated in SDS-PAGE and immunoblotted with an anti-PBX1 antibody. Full-length blots are included in Supplementary Fig. S5. (**b**) Diagram shows LAP-tagged E2A-PBX1 and the tandem affinity LAP purification scheme. (**c**) Eluates from LAP purifications using RCH-ACV cells stably expressing vector or LAP-tagged E2A-PBX1 were resolved by SDS-PAGE and silver stained. The three bands of E2A-PBX1 were from E2A-PBX1 degradation. (**d**) Co-immunoprecipitation assays show E2A-PBX1 interaction with PBX1, PBX2, and PBX3. His-tagged PBX1, PBX2, or PBX3 were co-transfected with vector only or Flag-tagged E2A-PBX1 into 293T cells. Cell lysates were immuno-precipitated with an anti-Flag antibody before western blotting with Flag and His antibodies. Full-length blots are included in Supplementary Fig. S5. (**e**) Results of cell proliferation assays are shown for RCH-ACV cells after control (shLuc) shRNA or shRNA-mediated knockdown of E2A-PBX1, PBX1, PBX2, or PBX3. Cell numbers were enumerated after 4 days and expressed relative to the numbers obtained with control shRNA-transduced cells. Data indicate means ± SEM (n = 3 independent experiments). Statistical analysis was performed by Student *t* test. ***p < 0.001; n.s., not significant.

To assess potential protein interactions of E2A-PBX1, gel filtration chromatography was performed using a whole cell extract prepared from the human lymphoblastic leukemia cell line RCH-ACV, which contains a t(1;19) chromosomal translocation^28^. E2A-PBX1 eluted at approximately 400 kD (Fig. 1a), much larger than its predicted monomeric mass of 90 kD, suggesting that it forms a higher-order complex. To identify candidate interacting proteins, we applied LAP (localization and tandem affinity purification) technology (Fig. 1b)^29^. LAP-tagged E2A-PBX1 was stably expressed in RCH-ACV cells and purified with GFP and Flag antibodies (Fig. 1b). Sixty co-purified proteins were identified by mass spectrometry in at least two of three independent experiments (Supplementary Fig. S1a). Among the identified spectra, the majority signals derived from E2A and PBX family proteins, including wild type PBX1, PBX2 and PBX3 (Supplementary Fig. S1a and Fig. 1c). Interaction of E2A-PBX1 with PBX family proteins was confirmed by co-immunoprecipitation (co-IP) assays (Fig. 1d). Although other proteins co-purified with E2A-PBX1 at lower abundance, we were unable to demonstrate their association with E2A-PBX1 under our co-IP conditions, including TCF12 and LMNB1, suggesting that they may be weak interactors of E2A-PBX1 or need other associating proteins to maintain stable interactions that otherwise may be disrupted during the co-IP process.

To gain insight into the molecular function of PBX homeodomain family proteins in E2A-PBX1 leukemogenesis, the respective wild type proteins were individually depleted in RCH-ACV cells. E2A-PBX1 depletion by PBX1 shRNA, which targets both E2A-PBX1 and wild type PBX1, significantly reduced and impaired cell growth whereas wild type PBX1 specific depletion (shPBX1-N) had very limited impact on cell growth (Fig. 1e and Supplementary Fig. S1b), consistent with the fact that PBX1 is expressed at very low levels (if at all) in B cell lineages^30^. Similarly, cell growth was not significantly affected following depletion of PBX2 or PBX3 alone or in combination (Fig. 1e and Supplementary Fig. S1b). Thus, despite the fact that E2A-PBX1 was capable of stably interacting with wild type endogenous PBX family proteins, their knockdown had no effect on cell growth in E2A-PBX1^+^ ALL cells. These findings raised the possibility that E2A-PBX1 may self-associate through the PBX1 portion of the chimera to form a tetramer (~400 kD) or higher-order oligomer in t(1;19) lymphoblastic leukemia cells.

### E2A-PBX1 self-associates through the PBX1 PBC-B domain of the chimeric protein

**Figure 2:**
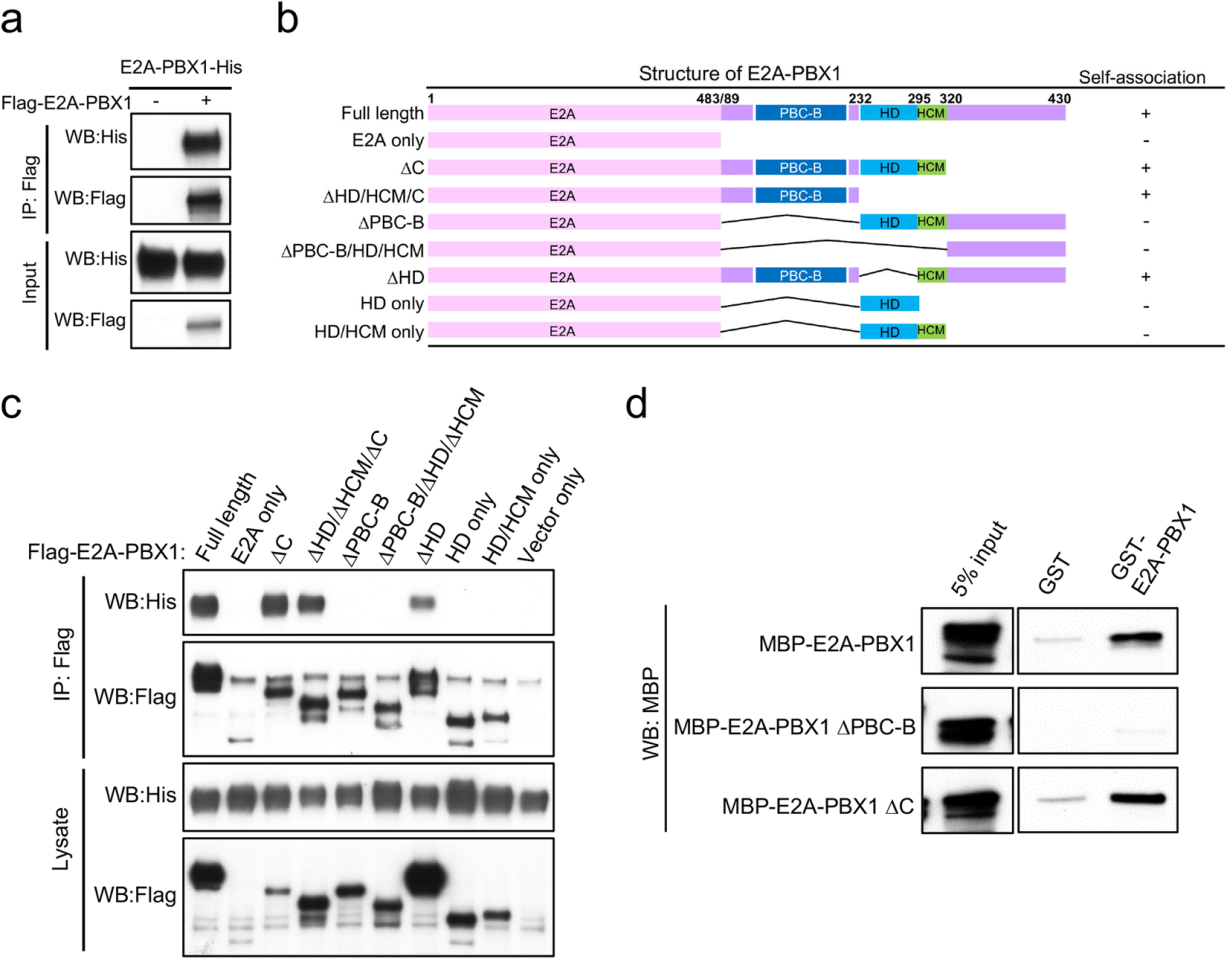
E2A-PBX1 self-associates through the PBX1 PBC-B domain of the chimeric protein. (**a**) Results are shown for co-immunoprecipitation assay in 293T cells co-transfected with His-tagged E2A-PBX1 and Flag-tagged E2A-PBX1. Cell lysates were immuno-precipitated with an anti-Flag antibody to demonstrate self-association of E2A-PBX1. (**b**) Schematic summary of E2A-PBX1 mutant constructs and their self-association abilities. HD, homeodomain; HCM, HOX cooperativity motif. (**c**) Flag-tagged mutant E2A-PBX1 proteins were co-transfected into 293T cells with His-tagged E2A-PBX1. Cell lysates were immuno-precipitated with an anti-Flag antibody to demonstrate that self-association of E2A-PBX1 requires the PBC-B domain of PBX1 in the chimeric protein. (**d**) GST-pull down assay shows that E2A-PBX1 self-associates directly through the PBC-B domain of PBX1. Full-length blots are included in Supplementary Fig. S5.

To investigate the oligomerization potential of E2A-PBX1, co-immunoprecipitation analyses were performed in 293T cells transiently co-expressing E2A-PBX1 proteins differentially tagged with either the Flag or His epitopes. Immunoprecipitation of Flag-tagged E2A-PBX1 with an anti-Flag antibody resulted in the co-precipitation of His-tagged E2A-PBX1 (Fig. 2a), demonstrating that E2A-PBX1 self-associates. Further analyses to localize the interaction site within E2A-PBX1 showed that mutants containing only the E2A portion did not interact with E2A-PBX1 (Fig. 2b,c), indicating that self-association occurs exclusively through the PBX1 portion of the chimeric protein consistent with the finding that E2A-PBX1 interacted with PBX family proteins in LAP purification. To further define regions of PBX1 responsible for self-association, a series of E2A-PBX1 deletion mutants was examined by co-immunoprecipitation analysis. These results showed that mutants with deletion of the PBC-B domain of PBX1 (ΔPBC-B) were unable to co-immunoprecipitate with E2A-PBX1 (Fig. 2b,c). All E2A-PBX1 mutant proteins localized to the nucleus excluding the possibility of localization changes (Supplementary Fig. S2). Direct protein-protein interaction assays using GST-pull down further confirmed that the PBC-B domain mediates E2A-PBX1 self-association (Fig. 2d).

### Self-association of E2A-PBX1 correlates with its oncogenic potential

**Figure 3:**
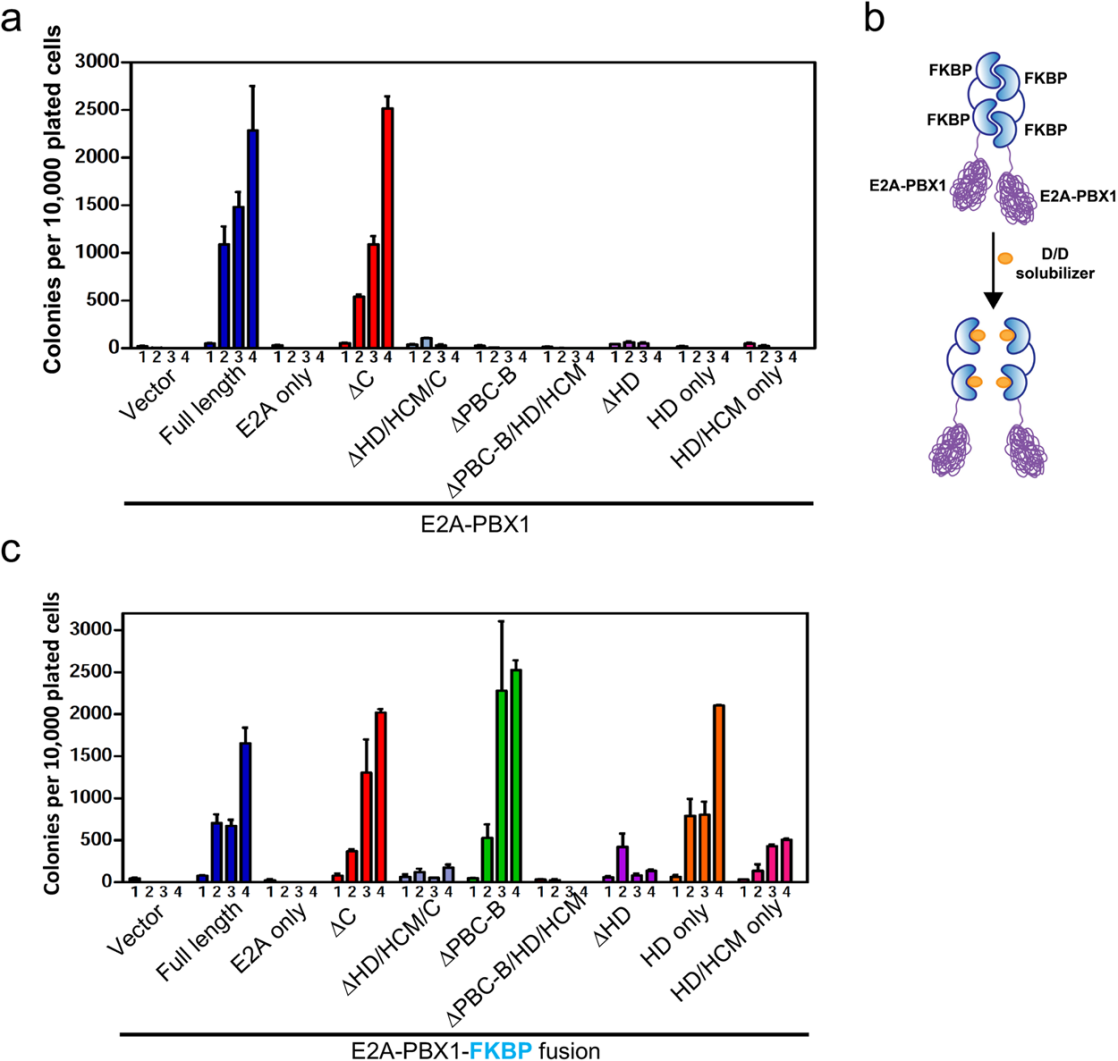
Immortalization of myeloid progenitors by E2A-PBX1 is dependent on its self-association. (**a**) Primary myeloid progenitors (c-kit^+^) were transduced with various E2A-PBX1 deletion constructs and assessed for their clonogenic potential through four rounds of serial plating in methylcellulose culture. Data indicate means ± SEM (n = 3 independent experiments). (**b**) Schematic illustration of conditional FKBP-mediated dimerization. (**c**) Primary myeloid progenitors (c-kit^+^) were transduced with various E2A-PBX1-FKBP fusion constructs and assessed for their clonogenic potential as in panel A. Data indicate means ± SEM (n = 3 independent experiments).

The role of self-association in E2A-PBX1 mediated oncogenic transformation was assessed using a methylcellulose serial replating assay that has previously been shown to reliably read out the transformative potential of E2A-PBX^31^. In contrast to intact E2A-PBX1, deletion mutants without the homeodomain (HD) were not able to sustain the clonogenic potential of myeloid progenitors in methycellulose cultures (Fig. 3a) suggesting that homeodomain-mediated DNA binding is indispensable for E2A-PBX1 oncogenic activity in this assay. Of note, mutants with PBC-B domain depletion (ΔPBC-B), even with intact homeodomain, lacked transformation ability, correlating completely with their inabilities to self-associate (Fig. 3a). This suggested that oligomerization may be a prerequisite for E2A-PBX1 oncogenesis. By contrast, the previously demonstrated abilities of these mutant proteins to bind DNA with the cooperation of HOX partners^22,32^, which depended on the extended PBX1 homeodomain containing the HOX cooperativity motif (HCM), did not correlate with transformation potential in this assay (Fig. 3a), suggesting that association of E2A-PBX1 with HOX partners alone is not sufficient for transformation.

To further assess the role of self-association, a set of E2A-PBX1 fusion proteins was constructed using a modified FK506-binding protein (FKBP) dimerization module^33^, which provides for conditional dimerization that can be disrupted using the synthetic agent D/D solubilizer (Fig. 3b). Two tandem copies of FKBP were fused in-frame to the carboxy termini of the various E2A-PBX1 deletion mutants (Fig. 3c). Fusion of full-length E2A-PBX1 to FKBP did not alter its ability to immortalize hematopoietic progenitors (Fig. 3c). However, the oncogenic potentials of several otherwise non-transforming E2A-PBX1 mutants were activated following fusion with FKBP as evidenced by their abilities to induce clonogenic growth of progenitors in the fourth round of methylcellulose culture (Fig. 3c) and establish cell lines in liquid culture (data not shown). The constructs that were not activated by FKBP lacked the PBX1 homeodomain, suggesting that forced oligomerization did not bypass a requirement for DNA binding in oncogenic transformation. In fact, the homeodomain was the minimal PBX1 requirement sufficient for transformation under these conditions as demonstrated by the robust ability of HD only-FKBP (Fig. 3c) to enhance clonogenic potential. One of the non-transforming constructs lacked all PBX1 sequences (E2A only) demonstrating that forced dimerization of only the E2A portion of the chimera was not oncogenic in the myeloid transformation assay (Fig. 3c). Taken together, these results suggest that oligomerization is required for transformation initiated by E2A-PBX1.

### Self-association is required for maintenance of E2A-PBX1-mediated transformation

**Figure 4:**
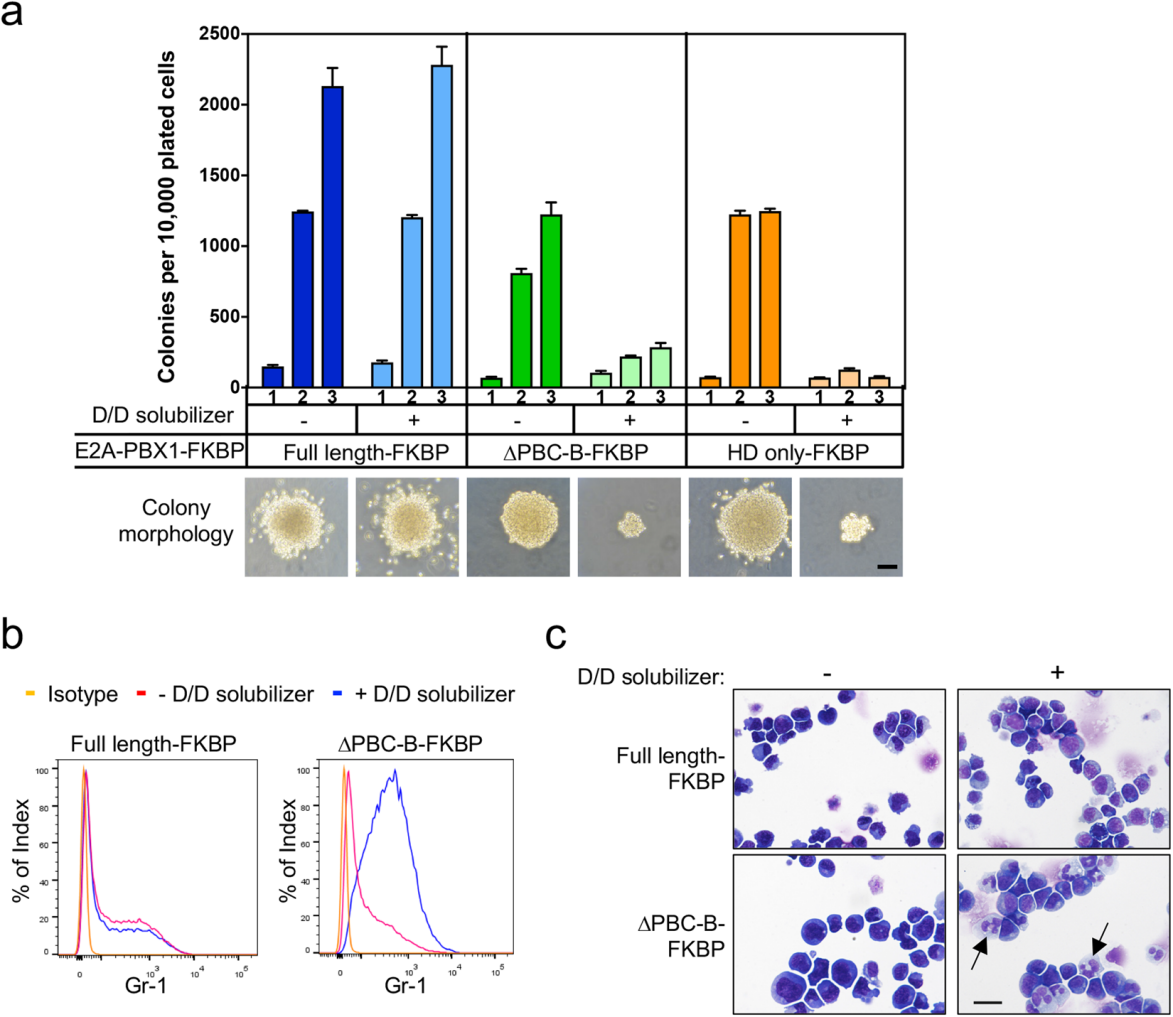
Self-association is required to maintain the oncogenic properties of E2A-PBX1 transformed cells. (**a**) Primary myeloid progenitors (c-kit^+^) were transduced with various E2A-PBX1-FKBP fusion constructs and assessed for their clonogenic potential through three rounds of serial plating in methylcellulose culture with or without D/D solubilizer. Representative colony morphologies are shown below the histogram. Data indicate means ± SEM (n = 3 independent experiments). Scale bar defines 200 μm. (**b**) FACS profiles show Gr-1 expression on E2A-PBX1-FKBP transformed cells cultured in medium with or without D/D solubilizer. (**c**) Cyto-preparations stained with May Grunwald-Giemsa solution show cells transformed with E2A-PBX1-FKBP fusions cultured in medium with or without D/D solubilizer. Representative images are shown. Scale bar defines 20 μm.

We next investigated the role for self-association in continued maintenance of E2A-PBX1-mediated transformation. The D/D solubilizer was added to methylcellulose culture medium to disrupt the artificial dimerization mediated by FKBP in the E2A-PBX1 mutants (Fig. 3b). When cells stably transformed by mutant E2A-PBX1-FKBP fusions were plated in methylcellulose culture in the presence of D/D solubilizer, their clonogenic potential was substantially impaired resulting in small colonies with diffuse morphology that failed to replate (Fig. 4a). This contrasted with the large blast-type colonies in the absence of drug, thus demonstrating a dependence upon FKBP-mediated oligomerization for maintaining enhanced self-renewal. By comparison, the clonogenic potential of cells transformed by full-length E2A-PBX1-FKBP was unaffected by D/D solubilizer presumably because self-association was mediated through the intact PBX1 moiety and not dependent on FKBP (Fig. 4a). In the absence of drug, the cultures initiated by PBC-B deleted mutant of E2A-PBX1-FKBP were comprised predominantly of Gr-1 low cells with the morphologic features of blasts (Fig. 4b,c). However, within 7 days of drug addition, the proportion of blasts was markedly reduced and the cultures contained mostly Gr-1 high cells with mature or maturing cytologic features (Fig. 4b,c). Taken together, these data demonstrate that the enhanced self-renewal, impaired differentiation, and sustained proliferation imposed by E2A-PBX1 are dependent on its ability to self-associate.

### Self-association of E2A-PBX1 confers its DNA binding ability

**Figure 5:**
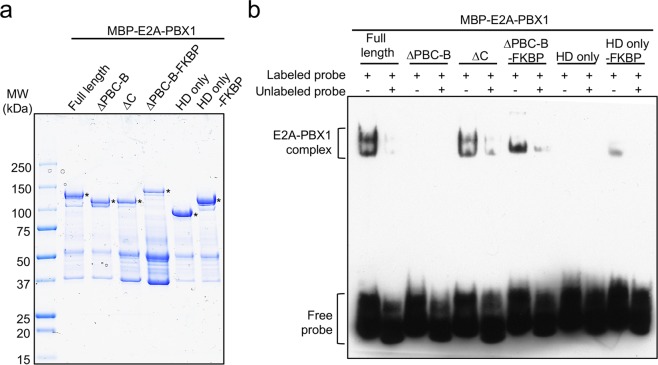
Self-association of E2A-PBX1 confers its DNA binding ability. (**a**) Coomassie blue stained SDS-PAGE gel shows purified MBP-tagged E2A-PBX1 recombinant proteins. MBP: maltose binding protein. *Intact protein. (**b**) Electrophoretic mobility shift assay was performed using a PBX1 consensus site with various recombinant E2A-PBX1 or E2A-PBX1-FKBP fusion proteins indicated at the top.

We hypothesized that the dependence on self-association in E2A-PBX1 transformation may be due to effects on DNA binding allowing E2A-PBX1 to cooperatively bind DNA with itself as a homo-dimer or oligomer. This may compensate for the inability of chimeric E2A-PBX1 to bind DNA as a monomer or hetero-dimerize with Meinox homeodomain protein partners, which otherwise cooperatively enhance wild type PBX1 DNA binding^34^. Consistent with this hypothesis, electrophoretic mobility shift assays using an oligonucleotide containing the consensus PBX1 site showed that the PBC-B deletion mutant of E2A-PBX1 lacked DNA binding ability but was rescued by fusion with FKBP (Fig. 5a,b). These data demonstrate that self-association activates E2A-PBX1 DNA binding, which correlates with its conditional transformation properties (Figs 3c and 4a).

### E2A-PBX1 self-association contributes to growth and maintenance of human E2A-PBX1^+^ preB-ALL

**Figure 6:**
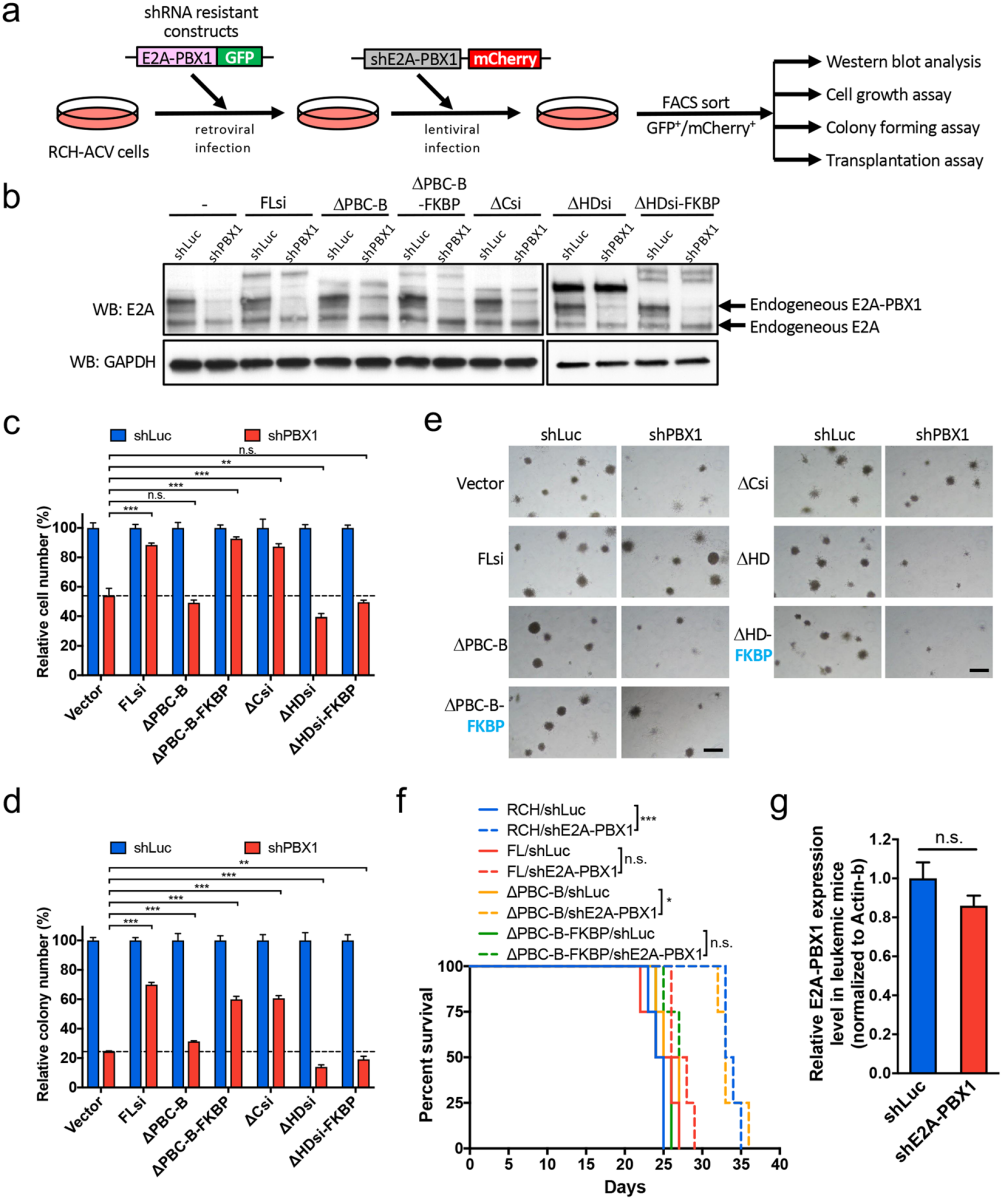
RCH-ACV cells are dependent on E2A-PBX1 self-association for growth and maintenance. (**a**) Experimental scheme for generating cells stably expressing shRNA-resistant exogenous E2A-PBX1 constructs for use in rescue experiments. (**b**) Western blot shows the levels of indicated proteins after E2A-PBX1 shRNA treatment of RCH-ACV cells expressing various shRNA-resistant E2A-PBX1 mutants. Endogenous E2A-PBX1 was efficiently depleted after shRNA treatment. “si” denotes E2A-PBX1 shRNA resistant constructs. Full-length blots are included in Supplementary Fig. S5. (**c**,**d**) Histograms show results of cell proliferation and colony-forming assays after shRNA-mediated knockdown of endogenous E2A-PBX1. Cell numbers (**c**) and colony numbers (**d**) were enumerated after 4 and 5 days, respectively, and expressed relative to the numbers obtained with control shRNA-transduced cells. Data indicate means ± SEM (n = 3 independent experiments). Statistical analysis was performed by Student *t* test. **p < 0.01; ***p < 0.001; n.s., not significant. (**e**) Representative colony morphologies are shown for experiment in (d). Scale bars define 400 μm. (**f**) Survival curves are shown for cohorts of mice transplanted with RCH-ACV cells stably expressing various E2A-PBX1 mutants and treated with control or E2A-PBX1 shRNAs (n = 4 in each cohort). Statistical analysis was performed by the Log-rank (Mantel-Cox) test. *p < 0.05; ***p < 0.001; n.s., not significant. (**g**) E2A-PBX1 transcript levels are shown for bone marrow cells isolated from diseased mice transplanted with RCH-ACV cells treated with control (shLuc) or E2A-PBX1 shRNAs. Data indicate mean ± SEM (n = 3). Statistical analysis was performed by Student *t* test. n.s., not significant.

To functionally delineate the role of E2A-PBX1 self-association in human E2A-PBX1^+^ leukemia cells, its potential oncogenic requirement was assessed using *in-vitro* and *in-vivo* transformation model systems. Various E2A-PBX1 mutant constructs that were resistant to shRNA-mediated PBX1 knockdown were expressed in RCH-ACV cells, which were then depleted of endogenous E2A-PBX1 by shRNA knockdown (Fig. 6a,b). Endogenous E2A-PBX1 depletion significantly reduced or impaired *in-vitro* growth of RCH-ACV cells in liquid culture or colony formation in methylcellulose medium (Fig. 6c–e). RCH-ACV cells expressing exogenous E2A-PBX1 full length (FLsi) and C-terminal deletion (ΔCsi) constructs resistant to shRNA knockdown continued to grow normally, despite the loss of endogenous E2A-PBX1 (Fig. 6c–e). In contrast, deletion mutants lacking the PBC-B domain (ΔPBC-B) or homeodomain (ΔHDsi) were unable to rescue growth of E2A-PBX1 depleted cells. However, fusion of the FKBP dimerization motif with the ΔPBC-B mutant, but not the ΔHD mutant, rescued cell growth and colony formation indicating that E2A-PBX1 self-association mediated by the PBC-B domain is required for RCH-ACV cell growth (Fig. 6c–e).

The requirement for E2A-PBX1 self-association in leukemogenesis was assessed *in vivo*. NOD-SCID mice were transplanted with RCH-ACV leukemia cells depleted of endogenous E2A-PBX1 and expressing various exogenous E2A-PBX1 constructs (Fig. 6f). Mice transplanted with E2A-PBX1-depleted cells survived longer than control mice and were not converted to shorter latency by expression of the exogenous PBC-B domain mutant (Fig. 6f). In contrast, mice transplanted with cells expressing the PBC-B domain deletion mutant fused with FKBP developed leukemia with a shortened latency comparable to control mice rescued by expression of full-length E2A-PBX1. Although leukemia eventually developed in mice transplanted with E2A-PBX1-depleted cells, this was caused by loss of E2A-PBX1 suppression by an unknown mechanism (Fig. 6g). Thus, self-association mediated by the PBC-B domain contributes to the leukemogenic properties of E2A-PBX1.

The critical requirement of self-association for growth and maintenance of E2A-PBX1^+^ preB-ALL was also assessed in a conditional E2A-PBX1 transgenic mouse model^35^. Mouse E2A-PBX1^+^ leukemia cells were first transduced with various E2A-PBX1 constructs resistant to shRNA-mediated knockdown and then depleted of endogenous E2A-PBX1 by shRNA knockdown. Transduced cells were then assessed in colony forming assays to evaluate the ability of exogenous constructs to rescue depletion of endogenous E2A-PBX1 (Supplementary Fig. S3a). The results were consistent with the foregoing studies using human RCH-ACV cells and confirmed that E2A-PBX1 self-association contributes to E2A-PBX1-induced preB-ALL (Supplementary Fig. S3b,c).

### Delineation of the self-association motif of E2A-PBX1

**Figure 7:**
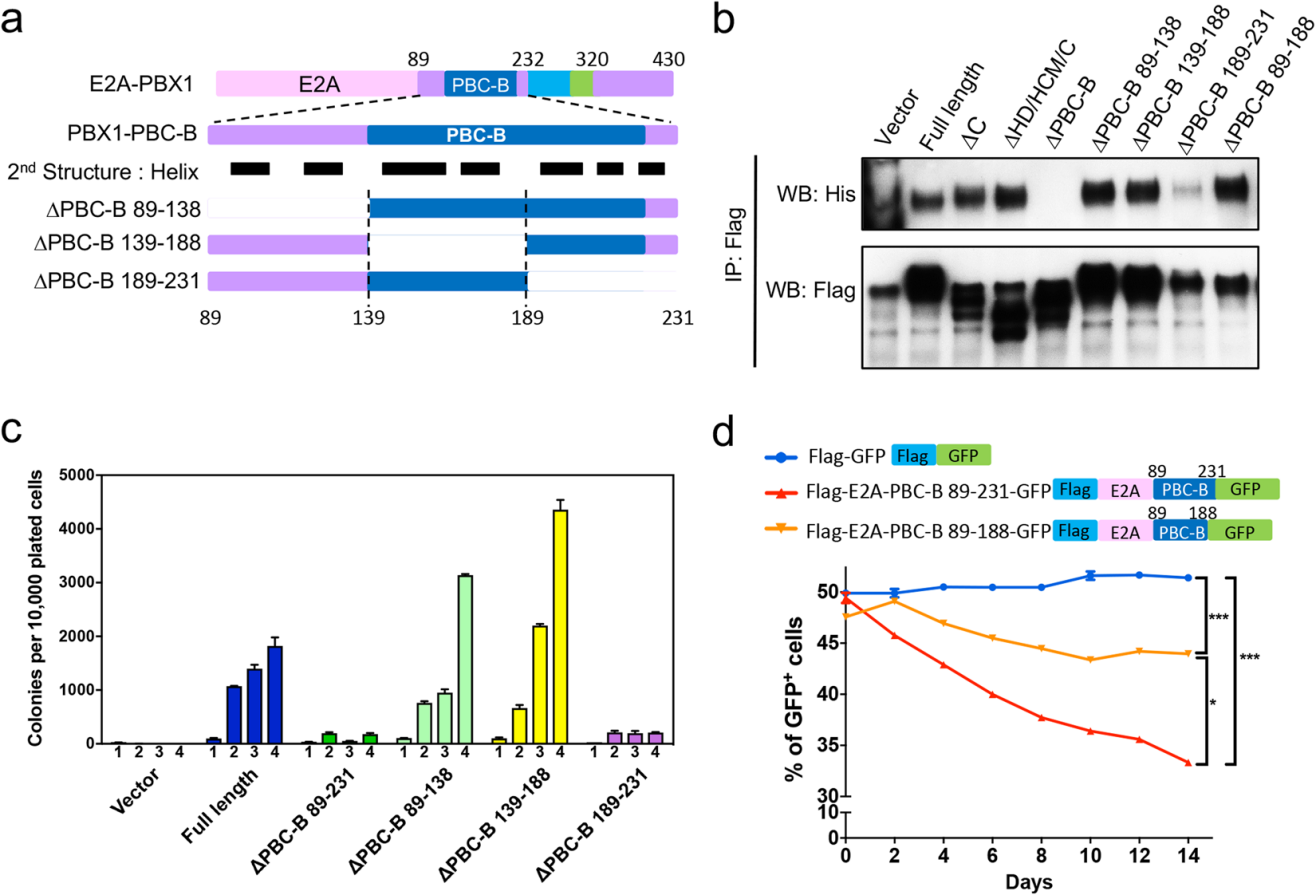
Delineation of the E2A-PBX1 dimerization motif. (**a**) Schematic illustration of PBC-B domain deletion mutants of E2A-PBX1. (**b**) Flag-tagged PBC-B domain mutants of E2A-PBX1 were co-transfected into 293T cells with His-tagged E2A-PBX1. Cell lysates were immuno-precipitated with an anti-Flag antibody to demonstrate that self-association of E2A-PBX1 requires the NC domain (aa 189–231) of PBX1. Full-length blots are included in Supplementary Fig. S5. (**c**) Primary myeloid progenitors (c-kit^+^) were transduced with various PBC-B domain deletion constructs and assessed for their clonogenic potential through four rounds of serial plating in methylcellulose culture. Data indicate means ± SEM (n = 3 independent experiments). (**d**) RCH-ACV cells were transduced with GFP only, GFP fusion E2A-PBX1-N (89–231 aa) or E2A-PBX1-N (ΔNC) (89–188 aa). GFP^+^ cells were sorted after 4 days, mixed 50/50 with non-treated RCH-ACV cells, and monitored every 2 days. Diagram shows mean ± SEM (n = 3 independent experiments). Statistical analysis was performed by F test. *p < 0.05; ***p < 0.001.

To further define the oligomerization motif in E2A-PBX1, additional PBC-B domain deletion mutants were generated based on its predicted secondary structure (Fig. 7a). Flag-tagged PBC-B domain mutants of E2A-PBX1 were co-transfected with His-tagged full-length E2A-PBX1 into 293T cells and co-immunoprecipitation was performed with an anti-Flag-antibody to evaluate self-association potential. A deletion mutant lacking amino acids 189–231 of PBX1 (ΔPBC-B 189–231) was unable to precipitate with His-tagged E2A-PBX1 (Fig. 7b), indicating that a small motif within the deleted region of PBC-B domain plays a key role mediating self-association of E2A-PBX1. Colony forming assays also showed that the ΔPBC-B 189–231 mutant of E2A-PBX1, similar to the ΔPBC-B mutant lacking amino acids 89–231, was unable to transform mouse myeloid progenitors (Fig. 7c) thereby defining a helical-rich motif in E2A-PBX1 required for self-association and oncogenesis.

We further tested whether the PBC-B domain may have dominant-negative effects on inhibiting cell growth when over-expressed in RCH-ACV cells. Indeed, RCH-ACV cells over-expressing the E2A-PBX1-PBC-B 89–231 mutant showed a 30% reduction of growth in liquid culture compared to control cells, while cells overexpressing the E2A-PBX1-PBC-B 89–189 mutant showed 10% reduction of growth (Fig. 7d), confirming that E2A-PBX1-PBC-B can associate with E2A-PBX1 but fail to bind DNA since HD domain is absent.

## Discussion

In this study, we demonstrate that self-association of the E2A-PBX1 chimeric transcription factor is indispensable for its oncogenic activity in human and mouse B-cell precursor leukemia cells, and primary myeloid progenitors. E2A-PBX1 self-associates through the PBX1 PBC-B domain to form a higher-order complex in t(1;19) lymphoblastic leukemia cells. A detailed structure/function analysis demonstrated the complete concordance of self-association with DNA binding, self-renewal, impaired differentiation, and sustained proliferation (Supplementary Fig. S4). Thus, our results from multiple experimental systems consistently support that self-association is crucial but not sufficient for E2A-PBX1 leukemia pathogenesis.

An oncogenic requirement for oligomeric self-association is a feature that E2A-PBX1 shares with several other chimeric transcription factors associated with acute leukemia pathogenesis^2^. For example, dimerization-induced co-repressor binding and relaxed DNA binding specificity are critical for PML-RARα-induced immortalization^36^, and forced RARα homodimers prime mice for leukemia^37^. Dimerization also activates the oncogenic properties of a subset of MLL fusion proteins (e.g. MLL-AF6) by promoting association with gene expression modulators^4,38–40^. In the case of AML1-ETO, the NHR2 domain, which mediates oligomerization and interactions with co-repressor molecules, is critical for AML1-ETO’s transcriptional and *in vitro* effects on myeloid differentiation and clonogenic potential^3,41^. Thus, a recurring scenario for oncogenic activation of chimeric transcription factors involves self-association, which serves to alter their transcriptional properties.

The mechanistic basis for the role of self-association in E2A-PBX1 oncogenesis appears to be operative at the level of DNA binding. This was demonstrated by the DNA binding ability and oncogenic activity of PBC-B domain deletion mutants artificially fused to FKBP. Intact E2A-PBX1 typically binds DNA poorly, if at all, as a monomer^32^. Furthermore, previous unbiased site selection screens for optimal PBX1 recognition sequences yielded tandem dimeric PBX1 core sequences (TGATTGAT) suggesting that the PBX1 homeodomain does not stably bind DNA as a monomer^42–44^. Thus, self-association may stabilize binding to PBX consensus sites by providing two or more homeodomains in trans. Indeed, forcing dimerization of E2A-PBX1 overrides the requirement of PBC-B domain and rescues the oncogenic activity of PBC-B deletion mutant of E2A-PBX1. However, we cannot rule out the possibility that self-association may also enhance or modulate the transcriptional effector properties of E2A-PBX1 mediated through the E2A moiety in addition to facilitating DNA binding. Our results also do not exclude potential heterologous protein interactions by E2A-PBX1, but suggest that they are likely too weak, unstable, or sub-stoichiometric to be identified using our approach.

Consistent with our results, previous studies have shown that the PBX1 DNA binding homeodomain is required for E2A-PBX1 oncogenic activity, underscoring the PBX1 target gene dependent character of E2A-PBX1-mediated transformation^22,45^. However, our results diverge from those of previous studies regarding the requirement for hetero-dimerization with HOX proteins. Some studies have suggested that it is necessary since the HCM domain of PBX1, which mediates HOX protein interaction, is required for fibroblast transformation *in vitro*, blocked differentiation of cultured murine myeloid progenitors, and acute myeloid leukemia in mice^22,24,27^. Although E2A-PBX1 oncogenicity in some of these assays is strongly enhanced by forced hyper-expression of HOXA9, HOX family genes are not consistently expressed in ALL cells^25,26^. Our studies suggest that hetero-dimerization with HOX proteins may be dispensable since the HCM deletion mutant retains oncogenic potential, and the homeodomain constitutes the minimum required portion of PBX1 for transformation induced by FKBP-mediated dimerization. Other studies differ from ours in not supporting a role for homo-dimerization of E2A-PBX1 in oncogenic activity^32,46^. Calvo *et al*. identified a 39 amino acid self-association motif (corresponding to PBX1 amino acids 168–206) within the PBC-B domain that partially overlaps with the motif identified in our study (189–231). However, the 39 amino acid motif was not required for myeloid immortalization *in vitro* by E2A-PBX1. Conversely, our studies demonstrate a requirement for self-association for E2A-PBX1 oncogenic activity in human and mouse B-cell precursor leukemia cell assays in addition to mouse myeloid transduction/transplantation assays. The observed divergence with previous studies may reflect differences in biological assays, or expression levels or compositions of constructs employed. Nevertheless, our results clearly indicate that self-association through the PBC-B domain and DNA binding through the homeodomain of PBX1 are the basic requirements for E2A-PBX1 oncogenic activity in B-lineage ALL cells and myeloid progenitors.

Our studies support a revised model for E2A-PBX1 mediated transformation. Fusion with E2A results in both gain- and loss-of-function effects on PBX1 transcriptional activity. The E2A moiety confers strong transcriptional activation and constitutive nuclear localization properties on E2A-PBX1, both of which are necessary for transformation. Conversely, as a consequence of PBC-A domain disruption, E2A-PBX1 lacks the ability to bind DNA or heterodimerize with Meinox homeodomain proteins^19,34^, which otherwise modulate PBX1 stability, nuclear localization, DNA binding, and transcriptional activity^7,20,21,47^. Nevertheless, E2A-PBX1 retains an ability to cooperatively bind DNA with HOX proteins as a simple heterodimer, which has led to suggestions that it may function with HOX partners to mis-regulate subordinate genes containing PBX/HOX binding sites. However, our current studies indicate that although HOX association ability of E2A-PBX1 is retained, it is not sufficient (Fig. 3). Rather, E2A-PBX1 must self-associate for transformation, which may compensate for the inability of monomeric E2A-PBX1 to bind DNA or heterodimerize with MEINOX homeodomain proteins.

Despite its key role in leukemia pathogenesis, E2A-PBX1 has not yet proven to be a “druggable” transcription factor for therapeutic targeting. Dependence of PBC-B domain mediated self-association for E2A-PBX1 oncogenic activity provides a rationale for its consideration as a therapeutic target for E2A-PBX1^+^ preB-ALL. A potential strategy is to disrupt E2A-PBX1 self-association. We further defined a minimum region in PBC-B domain required for E2A-PBX1 self-association. Small molecules designed to target this region to disrupt self-association merit further investigation.

## Materials and Methods

### Cell culture

293T and Phoenix cells were cultured in DMEM medium supplemented with 10% FBS, 100 U/mL penicillin/streptomycin, and 0.29 mg/mL L-glutamine. Human leukemia cell line RCH-ACV was cultured in RPMI1640 medium supplemented with 10% FBS, 100 U/mL penicillin/streptomycin, and 0.29 mg/mL L-glutamine. Mouse E2A-PBX1 leukemia cells were cultured as previously described^35^.

### Expression vector construction and retrovirus preparation

Plasmids encoding mutant forms of E2A-PBX1 have been reported previously^22^ or were constructed by PCR and standard cloning techniques in MSCV (for retroviral transductions, Clontech), pGEX-4T-1 (GE Healthcare), and pMAL-c2X (New England BioLabs) vectors. Two copies of the FKBP dimerization module were released by XbaI/BamHI digestion of Pc4-FM2E (Ariad Pharmaceuticals, Inc.) and fused in-frame to the carboxyl termini of various E2A-PBX1 constructs. For construction of PBX1 shRNA resistant constructs, a 5′ primer (GACAACTCAGTGGAGCAcagcGAcTAtAGgGCCAAACTCTCACAGATCAG, lower cases indicate mismatch nucleotides) with seven nucleotides mismatch to PBX1 shRNA targeted sequence and 3′ primer (CTCCACTGAGTTGTCTGAACC) were used for PCR amplification followed by InFusion cloning using InFusion Cloning Kit (Clontech laboratories Inc.). All constructs were confirmed by Sanger sequencing. Retrovirus generation and transduction of human leukemia cells are described elsewhere^48^. Briefly, retrovirus was packaged in Phoenix-Eco cells and RCH-ACV cells expressing ecotropic receptor were used for transduction by spinoculation (2,500 rpm, 32 °C for 2.5 hr).

### shRNA design, lentivirus generation and cell transduction

shRNAs were designed using a commercial web tool (Invitrogen). Individual shRNA sequences (shPBX1 (5′ GGAGCATTCAGATTACAGA 3′), shPBX1-N (5′ AGCCGAGGAGCAGAAGA GGAAG 3′), shPBX2 (5′ GGTATCCCAGGTCTCGGTTCA 3′), and shPBX3 (5′ GCAGCCTC TGGAGGTTCTTCA 3′)), were cloned into p309-mCherry lentiviral vector^49^. Lentivirus generation and transduction of human leukemia cells are described elsewhere^48^. The sorted mCherry^+^ cells were cultured for 4 days for cell proliferation assay or used in bone marrow transplantation experiments.

### Myeloid progenitor cell transformation assay

Myeloid progenitors were transduced using retroviral constructs essentially as described previously^50^ with minor modifications^38^. In brief, c-kit^+^ cells were selected from the bone marrow of 4–8 weeks old C57BL/6 mice using an auto-MACS and anti-c-kit magnetic beads (Miltenyi Biotech). Purified c-kit^+^ cells were transduced with retrovirus and plated in methylcellulose medium supplemented with cytokines (20 ng/ml SCF, and 10 ng/ml of IL-3, IL-6, and GM-CSF) (PeproTech) with appropriate drug selection. Colonies were counted 7 days after plating, after which cells were harvested, washed, and replated (10,000 cells per assay). After the fourth round of serial replating, transformed cells were cultured in R20/20 medium (RPMI1640 medium containing 20% WEHI conditioned medium and 20% FBS) to establish continuous cell lines. For experiments with FKBP fused constructs, D/D solubilizer (Clontech) was added where indicated to methylcellulose at a concentration of 4 µM.

### Flow cytometry

Cells were washed once with PBS, and then suspended in PBS containing 5% BSA. Antibody against Gr-1 (RB6-8C5 clone, BD Bioscience) was added to the cell suspension at 1:100 dilutions. After 20 min incubation on ice, cells were washed once, suspended in PBS with 5% BSA and analyzed using an LSR Model 1a flow cytometer (BD Biosciences).

### Gel filtration chromatography

Crude extract or elution fractions from Q Sepharose (250 mM KCl) or heparin Sepharose (200 mM KCl) were applied to a Superose 6 (3.2/30) column (Amersham Pharmacia Biotech) at a flow rate of 40 µl/min in buffer A (25 mM Tris-HCl, pH 8.5, 1 mM EDTA, 0.2% NP-40, 10% glycerol) containing 5 mM dithiothreitol, protease inhibitors and 200 mM KCl. Column fractions (50 µl) were collected and subjected to western blot analysis. Protein markers (Sigma) were eluted under the same condition to estimate molecular weights.

### Co-immunoprecipitation and immunoblot analysis

Two days after transfection of 293T cells with various plasmids, cells were harvested, washed with PBS, and suspended in Buffer A. Cells were lysed by passing 10 times through 26-G pestle before centrifugation (15,000 × g) for 10 minutes at 4 °C. Clarified protein extract was used either for immunoblot analysis or immunoprecipitation. Primary antibodies for western blot analyses consisted of anti-Flag (M2, Sigma), anti-His (sc-805, Genescript), anti-MBP (NEB), and anti-GAPDH (Sigma) antibodies. Monoclonal antibodies specific for PBX1 and E2A have been reported previously^19,51^. The blots were reacted with primary antibodies followed by peroxidase-conjugated secondary antibodies. Membranes were then incubated with ECL western blot detection kits (GE Healthcare) and images were detected by exposure to X-ray film.

### Transplantation assays

Transplantation assays were performed as previously described^48^. RCH-ACV human leukemia cells, stably expressing various E2A-PBX1 mutant constructs that are resistant to shRNA-mediated PBX1 knockdown, were transduced with control (shLuc) or E2A-PBX1 shRNA lentivirus with mCherry fluorescence reporter by spinoculation. After 72 hours, sorted mCherry^+^ cells in 0.2 mL PBS containing 0.5% FBS were transplanted into sublethally irradiated (200 rad) NOD.Cg-*Prkdc*^*scid*^ *Il2rg*^*tm1Wjl*^/SzJ (NSG) mice (8–10 weeks old).

### Electrophoretic mobility shift assays

Proteins used for DNA binding assays were purified using amylose resin according to users’ manual (New England BioLabs). DNA binding reactions were performed according to manufacturer’s instructions (LightShift Chemiluminescent EMSA kit, Thermo Scientific). Double-stranded oligonucleotides (TGATTGATTGATTGAT) used for DNA binding were end-labeled with biotin. Briefly, DNA binding reactions were carried out at room temperature for 20 min and the reaction mixtures were then subjected to electrophoresis using 5% TBE gels in 0.5X TBE buffer. After transferring to nylon membrane, the blots were reacted with streptavidin-horseradish peroxidase conjugate. Membranes were then incubated with ECL western blot detection solution and images were detected by exposure to X-ray film.

### LAP purification and mass spectrometry

Purification of LAP-E2A-PBX1 complexes was performed as described previously^29,52^ with some modifications. RCH-ACV cells expressing LAP-tagged fusion proteins were harvested and lysed in LAP150 buffer (50 mM HEPES, pH 7.4, 150 mM KCl, 1 mM EGTA, 1 mM MgCl_2_ 0.3% NP-40, 10% glycerol) containing protease inhibitors, followed by centrifugation at 100,000 × g for 1 hr. The resulting extract was incubated with protein A-coupled GFP antibody beads. After extensive wash, the beads were incubated with PreScission protease. The eluate was then incubated with anti-Flag M2 affinity gel (Sigma) and LAP-E2A-PBX1 complexes were eluted with Flag peptides (Sigma) and subjected to mass spectrometry analysis. Mass spectrometry, peptide identification, data processing and analysis were performed by the Vincent Coates Foundation Mass Spectrometry Laboratory at Stanford University Mass Spectrometry as described elsewhere^53^.

### Quantitative real-time PCR

RNA was isolated using TRIZOL Reagent and cDNA was synthesized using iScript Reverse Transcription Supermix (Bio-Rad) following the manufacturer’s recommendations. Quantitative PCR analysis was performed using a CFX384 real-time PCR system (Bio-Rad) with TaqMan Master Mix and primers (Applied Biosystems). All signals were quantified using ΔCt method and were normalized to the level of *Actb*. TaqMan probes from Life Technologies used in real-time PCR are Hs00231228_m1 (PBX1, detects E2A-PBX1 and wild type PBX1), Hs01114333_m1 (PBX1-N, detects wild type PBX1), Hs01901345_g1 (PBX2), and Hs00608415_m1 (PBX3).

### Study approval

All experiments on mice were performed with the approval of and in accordance with Stanford’s Administrative Panel on Laboratory Animal Care (APLAC, Protocol 9839).

## Supplementary information

Supplementary Information
