# Supplementary material for: Oligomeric self-association contributes to E2A-PBX1-mediated oncogenesis
Source: Sci Rep. 2019 Mar 20;9:4915. doi: 10.1038/s41598-019-41393-w (PMC6426973; doi:10.1038/s41598-019-41393-w)
Supplement: Supplementary file 1 — Supplementary Information [file 41598_2019_41393_MOESM1_ESM.pdf]

Oligomeric self-association contributes to E2A-PBX1-mediated oncogenesis

Chiou-Hong Lin, Zhong Wang, Jesús Duque-Afonso, Stephen Hon-Kit Wong, Janos Demeter, Alexander V. Loktev, Tim C. P. Somervaille, Peter K. Jackson and Michael L. Cleary

Supplementary Information

Supplementary Information

a

Protein ID	# of spectra	Protein ID	# of spectra	Protein ID	# of spectra
PBX1	428	RPL38	6	RFX1	3
PBX3	124	DDX5	6	IGLL1	3
TRAF4	34	RNF219	6	HSPA6	3
HNRNPF	32	RPL23	5	SORBS3	2
DDX17	25	IQSEC1	5	EIF4E2	2
PBX2	19	MAGED1	5	RPS9	2
BAG2	16	MASTL	5	ATP5C1	2
DNTT	16	RPS27L	4	PABPC1	2
PRPF39	13	LMNB1	4	TUBB4A	2
SEC16A	13	SPC24	4	TNKS	2
UBR5	13	TCF12	4	ARHGEF2	2
RBM14	11	MAD1L1	4	SEC13	2
EIF3D	11	UBAP2L	4	SKIV2L2	2
HNRNPH1	11	RICTOR	4	SMTN	2
GIGYF2	10	BRCA2	4	RPS27	1
AKAP8L	9	HAUS2	3	RPS2	1
AKAP8	9	EWSR1	3	DCAF7	1
NRIP1	8	YTHDF2	3	MAPKAP1	1
CBFA2T3	8	ATAD3B	3	PUM2	1
RCN2	7	LRMP	3	CRTC3	1

b

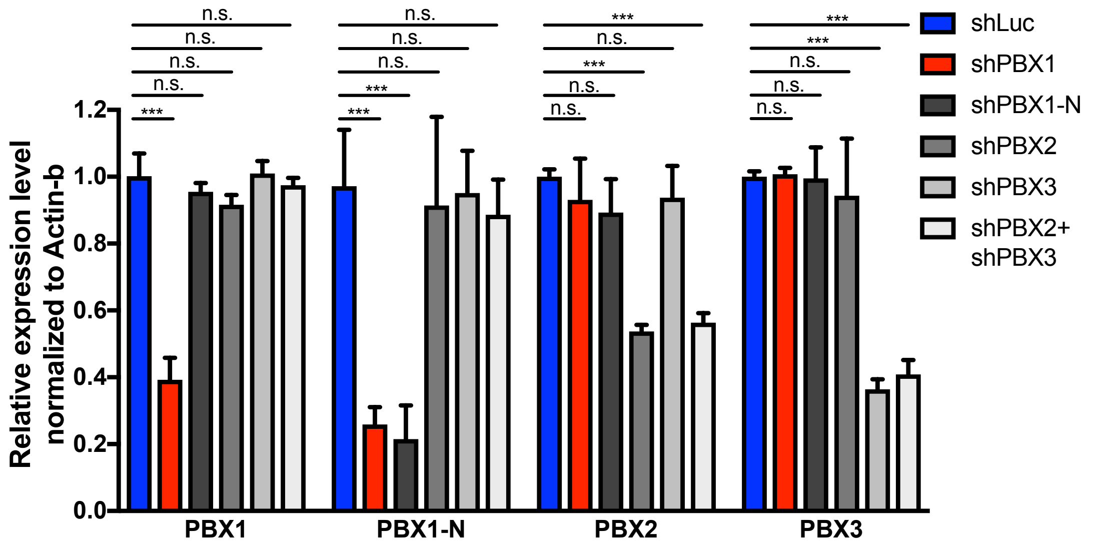

Supplementary Figure S1. Potential E2A-PBX1 interacting proteins identified from LAP purification.
(a) Sixty proteins were identified in at least two of three independent experiments. Number of spectra of each identified protein are shown.
(b) RCH-ACV cells were transduced with lentiviral vectors expressing control (shLuc), PBX1 (targets E2A-PBX1 and wild type PBX1), PBX1-N (targets wild type PBX1), PBX2, or PBX3 shRNAs. mCherry positive cells were sorted and relative transcript levels were quantified by qRT-PCR 4 days after transduction. Data indicate means ± SEM (n=3 independent experiments). Statistical analysis was performed by Student *t* test. n.s., not significant; ***, p<0.001.

Supplementary Figure S2

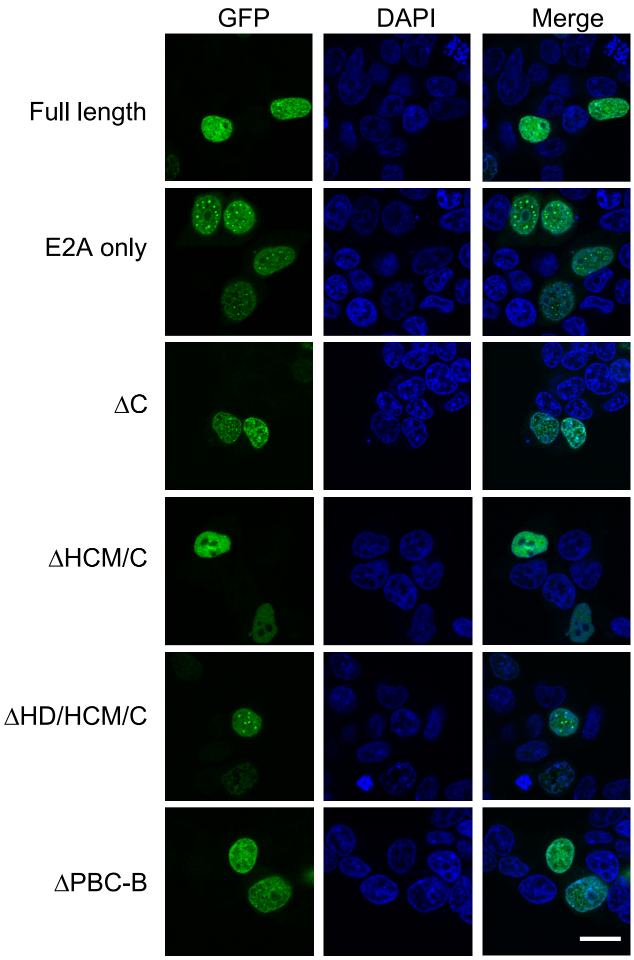

Supplementary Figure S2. E2A-PBX1 WT and mutants localize to the nucleus.
Representative images of 293T cells expressing various E2A-PBX1-GFP fusion mutants. Cells were stained with DAPI. Blue, DAPI (DNA); Green, E2A-PBX1-GFP fusion. Scale bar defines 10 μm.

Supplementary Figure S3

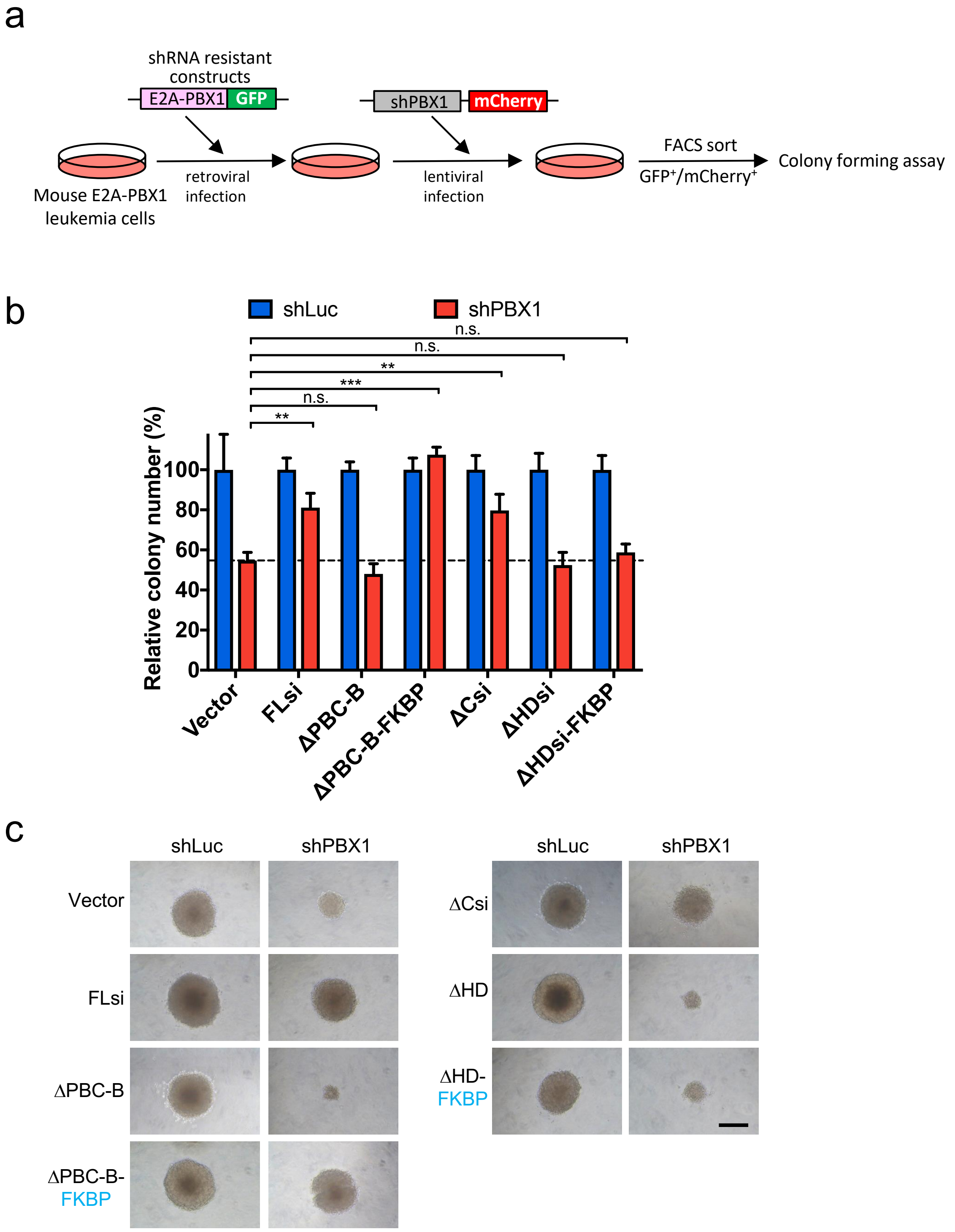

Supplementary Figure S3. Mouse ALL cells are dependent on E2A-PBX1 self-association for growth.
(a) Experimental scheme for generating ALL cells expressing shRNA-resistant E2A-PBX1 constructs for rescue of endogenous E2A-PBX1 knockdown.
(b) Histogram shows results of colony-forming assays after shRNA-mediated knockdown of endogenous E2A-PBX1. “si” denotes E2A-PBX1 shRNA resistant constructs. Data represent means ± SEM (n=3 independent experiments). Statistical analysis was performed by Student *t* test. **, $p < 0.01$; ***, $p < 0.001$; n.s., not significant.
(c) Representative colony morphologies are shown for experiment in (b). Scale bar defines 400 μm.

Supplementary Figure S4

Summary

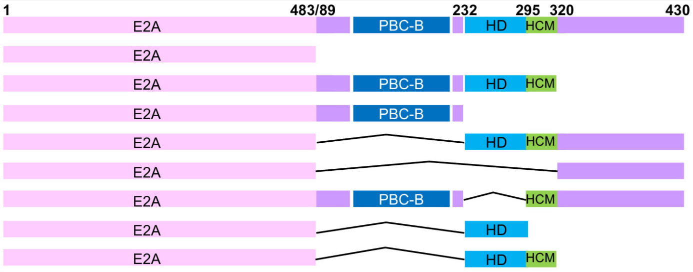

Structure of E2A-PBX1	Self-association	Myeloid transformation	DNA binding	FKBP fusion: Myeloid transformation - D/D	FKBP fusion: Myeloid transformation + D/D	FKBP fusion: DNA binding
Full length	+	+	+	+	+	+
E2A only	-	-	-	-	n.d.	n.d.
ΔC	+	+	+	+	+	+
ΔHD/HCM/C	+	-	-	-	n.d.	-
ΔPBC-B	-	-	-	+	-	+
ΔPBC-B/HD/HCM	-	-	-	-	n.d.	-
ΔHD	+	-	-	-	n.d.	-
HD only	-	-	-	+	-	+
HD/HCM only	-	-	-	+	-	+

Supplementary Figure S4. Summary of results.
Diagram summarizes the properties of various E2A-PBX1 proteins in functional assays of self-association, DNA binding, and leukemic transformation. HD, homeodomain; HCM, HOX cooperativity motif; FKBP, FK506-binding protein; n.d., not determined.

Supplementary Figure S5

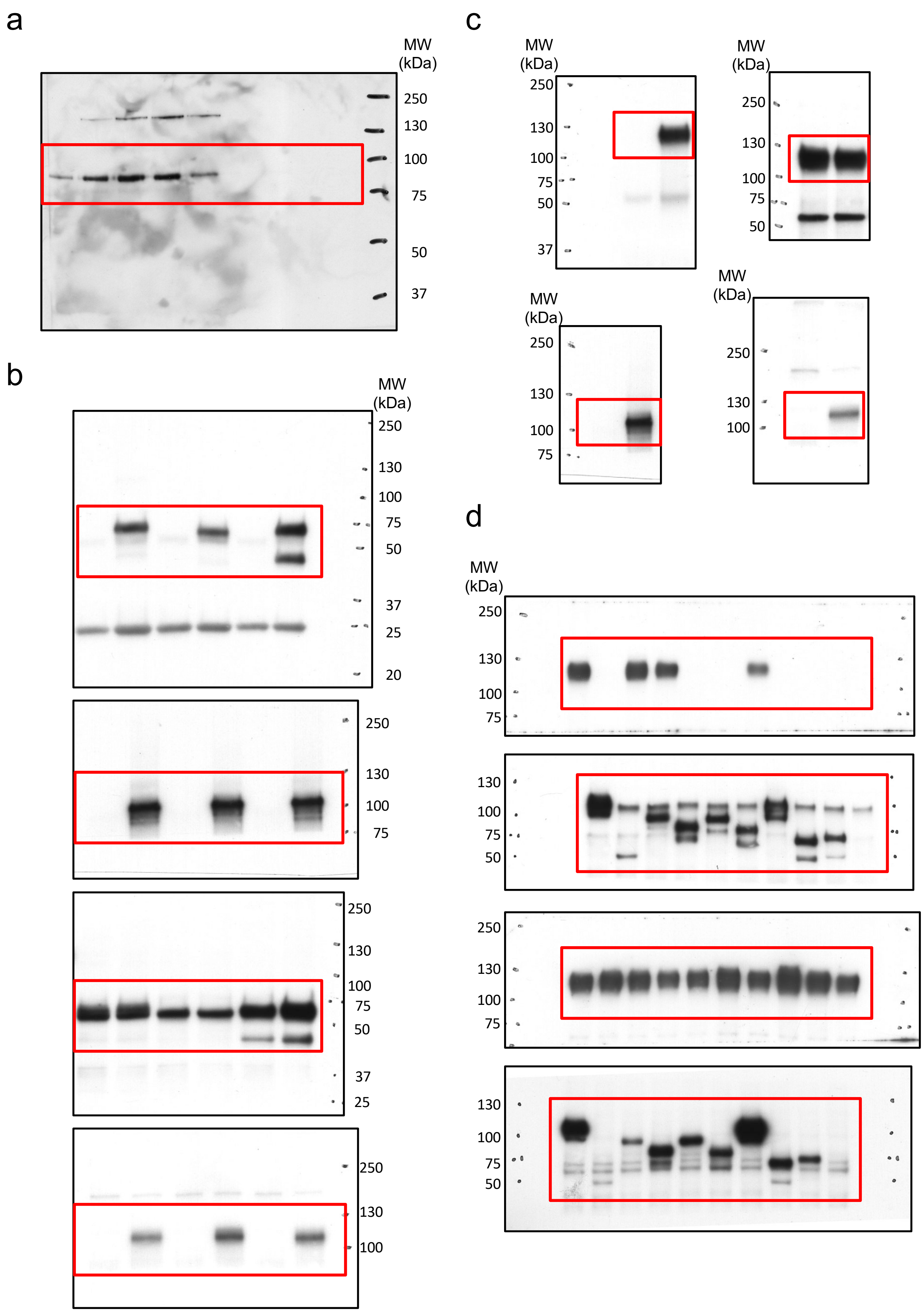

Supplementary Figure S5

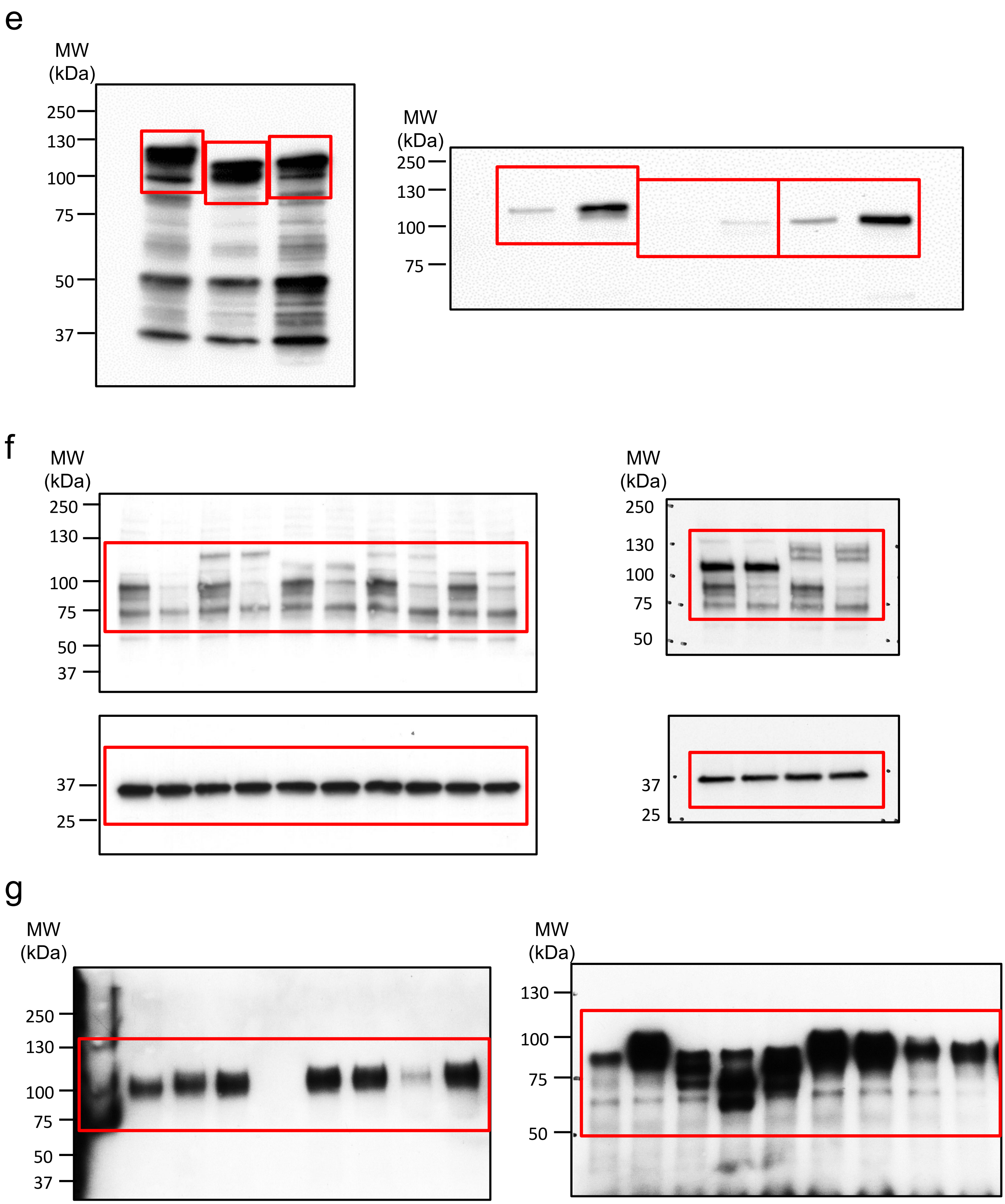

Supplementary Figure S5. Full-length blots related to Figures 1, 2, 6 and 7.
Portions that have been chosen for presentation are marked in red. (a) Full-length blots related to Fig. 1a. (b) Full-length blots related to Fig. 1d. (c) Full-length blots related to Fig. 2a. (d) Full-length blots related to Fig. 2c. (e) Full-length blots related to Fig. 2d. (f) Full-length blots related to Fig. 6b. (g) Full-length blots related to Fig. 7b.
